# Inhibition of the Soluble Epoxide Hydrolase Promotes Albuminuria in Mice with Progressive Renal Disease

**DOI:** 10.1371/journal.pone.0011979

**Published:** 2010-08-04

**Authors:** Oliver Jung, Felix Jansen, Anja Mieth, Eduardo Barbosa-Sicard, Rainer U. Pliquett, Andrea Babelova, Christophe Morisseau, Sung H. Hwang, Cindy Tsai, Bruce D. Hammock, Liliana Schaefer, Gerd Geisslinger, Kerstin Amann, Ralf P. Brandes

**Affiliations:** 1 Institut für Kardiovaskuläre Physiologie, Fachbereich Medizin der Goethe-Universität, Frankfurt am Main, Germany; 2 Medizinische Klinik III, Klinikum der Goethe-Universität, Frankfurt am Main, Germany; 3 Institute for Vascular Signalling, Klinikum der Goethe-Universität, Frankfurt am Main, Germany; 4 Department of Entomology and Cancer Center, University of California Davis, Davis, California, United States of America; 5 Pharmazentrum Frankfurt/ZAFES/Institut für Allgemeine Pharmakologie, Klinikum der Goethe-Universität, Frankfurt am Main, Germany; 6 Pharmazentrum Frankfurt/ZAFES/Institut für Klinische Pharmakologie, Klinikum der Goethe-Universität, Frankfurt am Main, Germany; 7 Department of Pathology, Nephropathology, Friedrich-Alexander University, Erlangen-Nürnberg, Germany; L' Istituto di Biomedicina ed Immunologia Molecolare, Consiglio Nazionale delle Ricerche, Italy

## Abstract

Epoxyeicotrienoic acids (EETs) are cytochrome P450-dependent anti-hypertensive and anti-inflammatory derivatives of arachidonic acid, which are highly abundant in the kidney and considered reno-protective. EETs are degraded by the enzyme soluble epoxide hydrolase (sEH) and sEH inhibitors are considered treatment for chronic renal failure (CRF). We determined whether sEH inhibition attenuates the progression of CRF in the 5/6-nephrectomy model (5/6-Nx) in mice. 5/6-Nx mice were treated with a placebo, an ACE-inhibitor (Ramipril, 40 mg/kg), the sEH-inhibitor cAUCB or the CYP-inhibitor fenbendazole for 8 weeks. 5/6-Nx induced hypertension, albuminuria, glomerulosclerosis and tubulo-interstitial damage and these effects were attenuated by Ramipril. In contrast, cAUCB failed to lower the blood pressure and albuminuria was more severe as compared to placebo. Plasma EET-levels were doubled in 5/6 Nx-mice as compared to sham mice receiving placebo. Renal sEH expression was attenuated in 5/6-Nx mice but cAUCB in these animals still further increased the EET-level. cAUCB also increased 5-HETE and 15-HETE, which derive from peroxidation or lipoxygenases. Similar to cAUCB, CYP450 inhibition increased HETEs and promoted albuminuria. Thus, sEH-inhibition failed to elicit protective effects in the 5/6-Nx model and showed a tendency to aggravate the disease. These effects might be consequence of a shift of arachidonic acid metabolism into the lipoxygenase pathway.

## Introduction

Epoxyeicosatrienoic acids (EETs) are anti-inflammatory derivatives of arachidonic acid (AA) which are generated by cytochrome P450 (CYP) epoxygenases [1]. EETs are antihypertensive, anti-inflammatory, anti-proliferative and pro-fibrinolytic. They act as an endothelium-derived hyperpolarizing factor (EDHF) in some vascular beds [1]. The CYP450 expression in the kidney is high and EETs promote renal sodium excretion [1], [2]. EET levels are dependent on the activity and expression of the CYP epoxygenases, which generate them and the enzyme soluble epoxide hydrolase (sEH) which converts the EETs to their corresponding dihydroxyeicosatrienoic acids (DHETs) [3]. DHETs subsequently leave the cell, can be conjugated in the liver and be excreted by liver or kidney [2], [4]. The activity of the sEH is therefore thought to be a major determinant of EET bioavailability [4]. Genetic deletion of the sEH as well as pharmacological inhibition increase plasma EET levels and potentiate their effects [5], and thus sEH inhibition elicits anti-hypertensive and anti-inflammatory effects [2], [5], [6]. Indeed, we have previously shown that sEH inhibition reduces angiotensin II-induced hypertension [7], neo-intima formation in hyperlipidemic mice [8] and vascular remodelling in the monocrotaline-model in rats [9].

Hypertension and inflammation are important progression factors for renal disease and thus it is logical to assume that sEH inhibition is a strategy to prevent progression of renal diseases [2], [5]. Indeed, it has been demonstrated that sEH inhibition improves renal vascular function, decreased glomerular injury and renal inflammation in rat models of angiotensin-induced and DOCA-salt hypertension [10]–[12]. A main limitation of these models is however that their high inflammatory activity does not necessarily reflect the situation of chronic renal disease in man which is dominated by sclerotic and fibrotic processes and which is characterized by a progressive, self-perpetuating nature [13], [14]. In animal experiments such a situation can be modelled by 5/6-nephrectomy (5/6-Nx). In this remnant kidney model, the substantial reduction in renal mass leads to compensatory renal hypertrophy, glomerula hyperfiltration and subsequently progressive chronic renal failure as consequence of glomerulo-sclerosis and interstitial fibrosis [15]–[17]. Although also in the remnant kidney model, the renin-angiotensin-system is involved in disease progression [18], it is only one of several factors contributing to a complex disease scenario.

Given the similarities between the remnant kidney model in rodents and the pathophysiology of progressive chronic renal failure in humans, we postulated that sEH inhibitors could be of therapeutic value. We tested this hypothesis in the rodent remnant kidney model. Unexpectedly and in contrast to previous data from inflammation driven renal failure models we observed that sEH inhibition had a tendency to accelerate the disease process in this model.

## Methods

### Animal preparations

SV129 which were purchased from Charles Rivers Laboratories (Sulzfeld, Germany) were used for this study, as other strains do not develop progressive renal failure mice [19]. Animals were housed in cages at constant temperature (22°C) and humidity (50%) and were exposed to a 12-hour dark/light cycle. Food and water were supplied ad libitum. The experiments were performed in accordance with the National Institutes of Health Guidelines on the Use of Laboratory Animals. Both, the University Animal Care Committee and the Federal Authorities for Animal Research of the Regierungspräsidium Darmstadt (Hessen, Germany) approved the study protocol (approval number V54-19c20/15-F28/05 and -F61/16). After 7 days of adaptation, the animals were randomly allocated to 5/6 nephrectomy (5/6-Nx) or sham operation. The surgery was performed under Isoflurane anaesthesia as previous described by others with modifications [19]. In brief: A left dorsal longitudinal incision was performed to expose the left kidney. The upper branch of the left renal artery was ligated by 6–0 prolene suture to produce about one third area with visible renal ischemia infarct; the lower pole of the left kidney (about one third kidney size) was removed by cautery. After 7 days of recovery, the right kidney was exposed in a similar preparation and removed after decapsulation and ligation of the vessels and the ureter to induce a total 5/6 nephrectomy (5/6-Nx). The control animals were sham operated in parallel by decapsulating the kidney. Early mortality within the first 3 days was approx. 20%. Subsequently, animals were randomized to the different treatment groups (n = 16 per group). The substances were administered as follows: The sEH inhibitor *cis*-4-[4-(3-adamantan-1-yl-ureido)-cyclohexyloxy]-benzoic acid (cAUCB, final concentration 8 mg/l), the ACE-inhibitor ramipril (final concentration 40 mg/l) were given with the drinking water, fenbendazole with the chow (100 mg/kg). Ramipril was kindly provided by Sanofi-Aventis, fenbendazole was from the university of Mainz (Zentrale Versuchstiereinheite - ZVTE) and cAUCB was synthesized by one of the coauthor as previously reported [20].

### Blood pressure measurements

Systolic blood pressure was assessed at 4 and 8 weeks after initiation of treatment. By an automated tail-cuff Blood Pressure Monitor (Visitech) in conscious, trained mice at room temperature as reported previously [7]. In a subset of animals blood pressure measurements were confirmed by telemetry with the aid of the data science instruments (DSI) system with the catheter being placed in the right iliac artery.

### Analysis of kidney function

Mice were placed in metabolic cages (Tecniplast) for 24-hour for urine collection. Urinary albumin was determined using the albumin-to-creatinine ratio (ACR). Urinary albumin and creatinine levels were measured by ELISA (Bethyl Laboratories, Montgomery, USA) and a creatinine assay kit (Labor+Technik, Berlin, Germany), respectively.

### Histological analysis

After the induction of terminal anesthesia, the abdominal vessels were opened, the thoracic cavity was opened, a canula was inserted into the right ventricle and the animals were perfused with phosphate-buffered saline (PBS). After the perfusion, kidneys were removed and fixed with 4% paraformaldehyde/PBS solution. The samples were subsequently embedded in paraffin and 2-µm sections were cut and stained with haematoxylin/eosin (HE), perjodic acid Schiff (PAS) and Sirius red (fibrous tissue stain). Thereafter, the stained kidney sections were analyzed by morphometry and stereology by investigations blinded to the study protocol. The following parameter were determined as reported previously [21]: To quantify mesangial matrix accumulation and sclerosis of the glomerular tuft, a score of 0 to 4 was determined on PAS and HE stained paraffin sections (GSI  =  Glomerulosclerosis index). A score of 0 was assigned for normal glomerulus, a score of 1 indicated mesangial expansion or sclerosis involving up to 25% of the glomerular tuft, a score of 2 indicated sclerosis of 25 to 50%, a score of 3 described sclerosis 50 to 75% and/or segmental extracapillary fibrosis or proliferation, and a score of 4 indicated global sclerosis >75%, global extracapillary fibrosis or complete collapse of the glomerular tuft.

The mesangiolysis score (MSI  =  Mesangiolysis index) was determined in PAS-stained paraffin sections and graded in 100 systematically subsampled glomeruli per animal using the following scoring system: score 0: no changes of capillaries, score 1: capillary dilatation <25% of the capillary convolute, score 2: capillary dilatation >25% of the capillary convolute or capillary aneurysms <50% of the capillary convolute, score 3: capillary aneurysms comprising 50–75% of the capillary convolute, score 4: capillary aneurysms comprising >75% of the capillary convolute [22].

Tubulointerstitial changes, i.e. tubular atrophy, dilatation, interstitial inflammation and interstitial fibrosis, and vascular damage, i.e. wall thickening and necrosis of the vessel wall, were assessed on HE stained paraffin sections as described at a magnification of 100× using a similar semi-quantitative scoring system from 0–4 [23]. In brief, for the determination of the tubulointerstitial damage, 10 fields per kidney were randomly sampled and graded as follows: grade 0 – normal tubulointerstitial structure; grade 1 – lesions involving less than 25% of the area; grade 2 – lesions affecting 25 to 50%; grade 3 – lesions involving more than 50% up to 75% and grade 4 with tubulointerstitial damage in almost the entire area (TSI  =  Tubulointerstitial damage index). Similarly, for the vascular damage score interlobular and smaller arteries were graded according to the following scheme: grade 0 – no wall thickening; grade 1, 2, 3 – mild, moderate and severe wall thickening, respectively; grade 4 – fibrinoid necrosis of the vascular wall.

### Determination of Arachidonic acid metabolites by liquid chromatography/tandem mass spectrometry

Blood samples were collected at the end of the experiment. After coagulation and centrifugation, the serum was stored at −80°C. At a later time point, samples were spiked with deuterated internal standards (5-HETE-d8, 12-HETE-d8, 15-HETE-d8, 20-HETE-d6, 8,9-EET-d8, 11,12-EET-d8 and 14,15-EET-d8) and extracted twice with ethyl acetate (0.5 ml). The sEH assay was extracted similarly but here one-tenth of organic phase was used and spiked. After evaporation of the ethyl acetate in a vacuum block under a gentle stream of nitrogen, the residues were reconstituted with 50 µl of methanol/water (1∶1, v/v) and analyzed with a Sciex API4000 mass spectrometer (AME Bioscience, Toroed, Norway) operating in multiple reaction monitoring (MRM) mode as described in detail elsewhere [24]. Chromatographic separation was performed on a Gemini C18 column (150×2 mm inner diameter, 5 µm particle size, Phenomenex, Aschaffenburg, Germany).

### Immunoblotting

Kidney samples were mortared in liquid nitrogen. For western blot analysis, cytosolic kidney protein (100 000 g supernatant, 20 µg) was boiled in Laemmli buffer, separated on sodium dodecyl sulfate–polyacrylamide gel electrophoresis (SDS–PAGE; 8%) and transferred onto nitrocellulose membrane as described (102). Proteins were detected using antibodies against sEH (provided by one of the coauthors) and glyceraldehyde 3-phosphate dehydrogenase (GAPDH/Santa Cruz Biotechnology, Santa Cruz, California, USA). Proteins were detected using appropriate secondary antibodies labeled with infrared dyes and visualized using the Odyssey infrared imaging system (Li-COR Biosciences, Bad Homburg, Germany) system. Densitometry was carried out using the integrated odyssey software.

### Real-time quantitative reverse transcription PCR

Organs were removed, flash frozen in liquid nitrogen and disrupted by grounding with mortar and pestle. RNA isolation was done with Absolutely RNA Miniprep Kit (Stratagene), cDNA synthesis with SuperScript III Reverse Transcriptase (Invitrogen) and random hexamer primers, semiquantitative real-time PCR with ABsolute QPCR SYBR Green Mix and ROX as reference dye (Thermo Scientific) in Mx4000 (Stratagene) with appropriate primers. For primer sequences please see table 1. Relative expressions of target genes were normalized to eukaryotic translation elongation factor 2 (EEF2), analyzed by delta-delta-Ct method and given as percentage compared to control experiments. PCRs for Cyp2c genes were done without SYBR Green but with fluorescent labelled probes.

**Table 1:** Primers for qRT-PCR.

ALOX5	forward	5′-CGGCTTCCCTTTGAGTATTGATGC-3′
	reverse	5′-CAGGAACTGGTAGCCAAACATGAG-3′
ALOX12	forward	5′-GGTTCTGCAACCTCATCACAGTTC -3′
	reverse	5′-CCAGCAGTAGGTCTGTTGTCTTTC -3′
ALOX15	forward	5′-GACACTTGGTGGCTGAGGTCTTTG-3′
	reverse	5′-GCTCCAGCTTGCTTGAGAAGATCC-3′
PLA2G4A	forward	5′-GTTCTACGTGCCACCAAAGTAACC-3′
	reverse	5′-TCCATGACGTAGTTGGCATCCATC-3′
Cyp2c38	forward	5′-CCCACTCCTTTCCCGATTAT-3′
	reverse	5′-CAGAAAACTCCTCCCCATGA-3′
	probe	5′-CY5-ATCAGAGCTTCCTTCACTGCTTCATACCCA-3′
Cyp2c40	forward	5′-AGGTCCAGCGGTACATTGAC-3′
	reverse	5′-CACAAATCCGTTTTCCTGCT-3′
	probe	5′-FAM-TTCATCCTCAAGGGAACACAGGTAA-3′
Cyp2c44	forward	5′-CAAAAAGGCTTGGTGGTGTT-3′
	reverse	5′-CCACAGATGGCCAAATTCTC-3′
	probe	5′-FAM-TTACATCGACTGTTTCCTCAGCAAGAT-3′
EEF2	forward	5′-GACATCACCAAGGGTGTGCAG-3′
	reverse	5′-GCGGTCAGCACACTGGCATA-3′
EPHX2	forward	5′-GAACATGAGTCGGACTTTCAAAAGCTTCTTC-3′
	reverse	5′-CCACAGTCCTCAATGTGTCCCCTTTTCAGG-3′

### Data and statistical analysis

All values are mean ± SEM. Statistical analysis was performed using analysis of variance (ANOVA); ANOVA followed by Bonferroni-corrected Fisher's LSD test, respectively, or, wherever appropriate, using a paired or unpaired t-test. A P value less than 0.05 was considered statistically significant.

## Results

### sEH inhibition does not prevent hypertension development and augments proteinuria in the remnant kidney model

**Figure 1:**
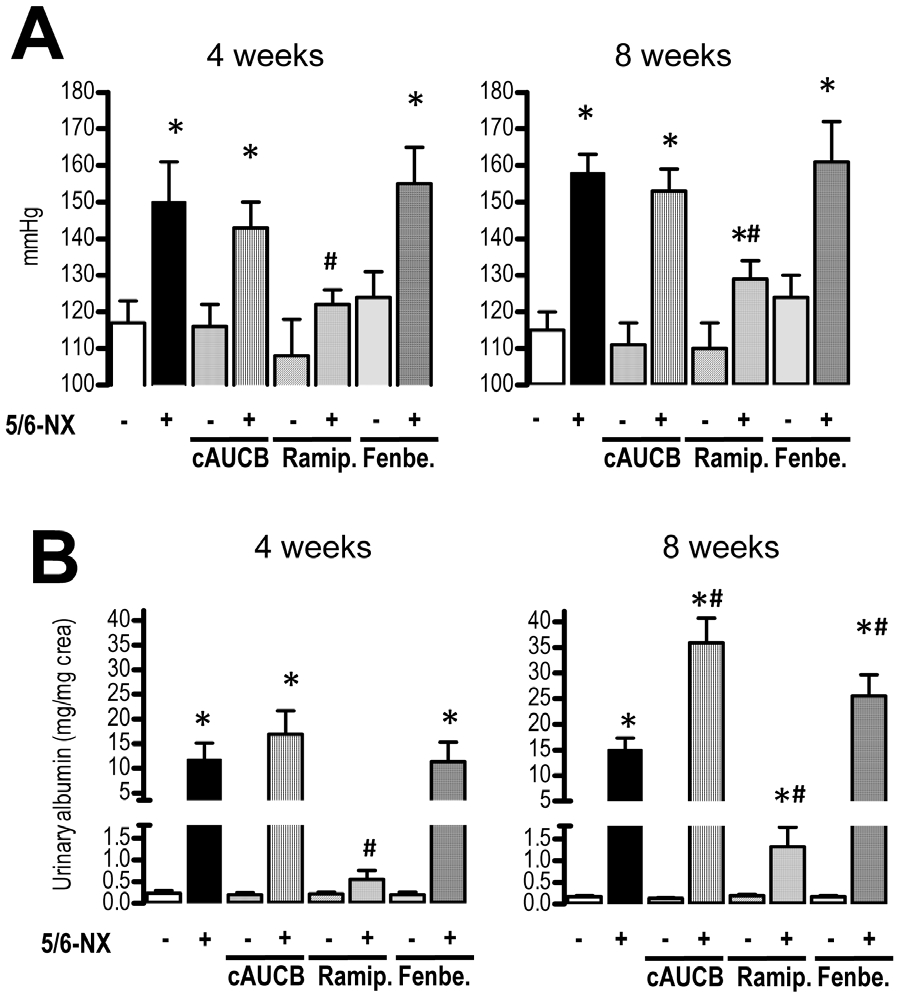
Blood pressure and albuminuria. Blood pressure measured by tail cuff technique (A) and albuminuria (B) were determined 4 and 8 weeks after initiation of treatment. Effects of placebo, sEH-inhibition by cAUCB, ACE-inhibition by ramipril (Ramip.) and CYP-inhibition by fenbendazole (Fenbe.) are shown on sham operated (−) and 5-6-Nx (+) animals. * p<0,05 vs. sham, # p<0,05 vs. placebo (n = 10–12/group).

As compared to sham operated mice, 5/6-Nx induced systemic hypertension present 4 weeks after the operation (p<0.0.5) that was maintained until the end of the observation period. Surprisingly, sEH-inhibition by cis-4-[4-(3-adamantan-1-yl-ureido)-cyclohexyloxy]-benzoic acid (cAUCB) had no effect on the development of hypertension, while ACE-inhibition by Ramipril significantly attenuated the process in 5/6-Nx mice at both time points (**Fig. 1A**). Tail-cuff measurements were verified by telemetry in a subset of animals (blood pressure in placebo treated animals: systolic blood pressure in sham-operated mice was 114±9 mmHg after 4 weeks and 112±4 mmHg after 8 weeks as compared to 169±11 mmHg after 4 weeks and 168±4 mmHg after 8 weeks in 5/6-Nx; diastolic blood pressure in sham-operated mice was 88±8 mmHg after 4 weeks and 87±5 mmHg after 8 weeks as compared to 128±9 mmHg after 4 weeks and 125±3 mmHg after 8 weeks in 5/6-Nx).

Albuminuria was determined after 4 and 8 weeks to assess renal damage (**Fig. 1B**). 5/6 nephrectomy induced marked albuminuria present at 4 weeks which was further increased 8 weeks after the operation. As expected, ACE-inhibition significantly attenuated the development of albuminuria. In contrast, sEH inhibition augmented albuminuria development in 5/6-Nx mice. While this effect was not statistically significant 4 weeks after operation, urinary albumin excretion after 8 weeks was 2-fold higher in the sEH inhibitor treated group as compared to placebo-treated animals (p<0.05).

### sEH inhibition does not prevent progression of renal damage

**Figure 2:**
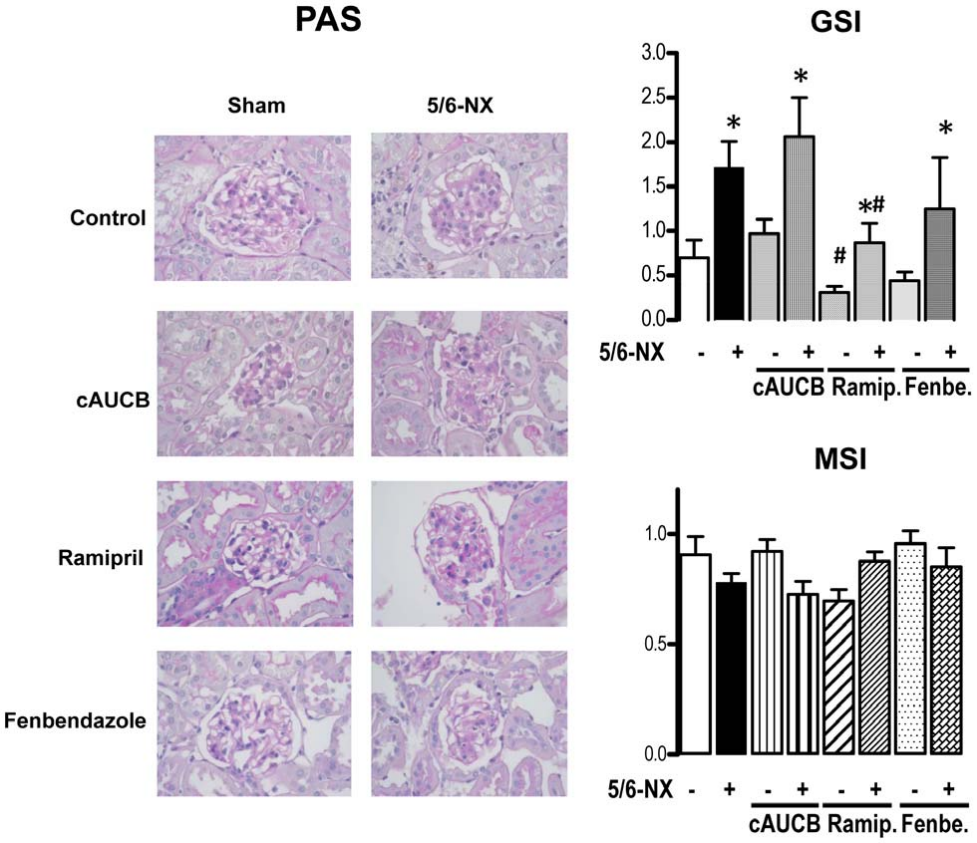
Histological glomerular changes. Representative kidney sections (magnification ×400) stained by periodide acid - Schiff (PAS) scoring glomerulosclerosis index (GSI) and mesangiolysis score (MSI) after 8 weeks of treatment. Effects of placebo, sEH-inhibition by cAUCB, ACE-inhibition by ramipril (Ramip.) and CYP-inhibition by fenbendazole (Fenbe.) are shown on sham operated (−) and 5-6-Nx (+) animals. * p<0,05 vs. sham, # p<0,05 vs. placebo (n = 5–8/group).

**Figure 3:**
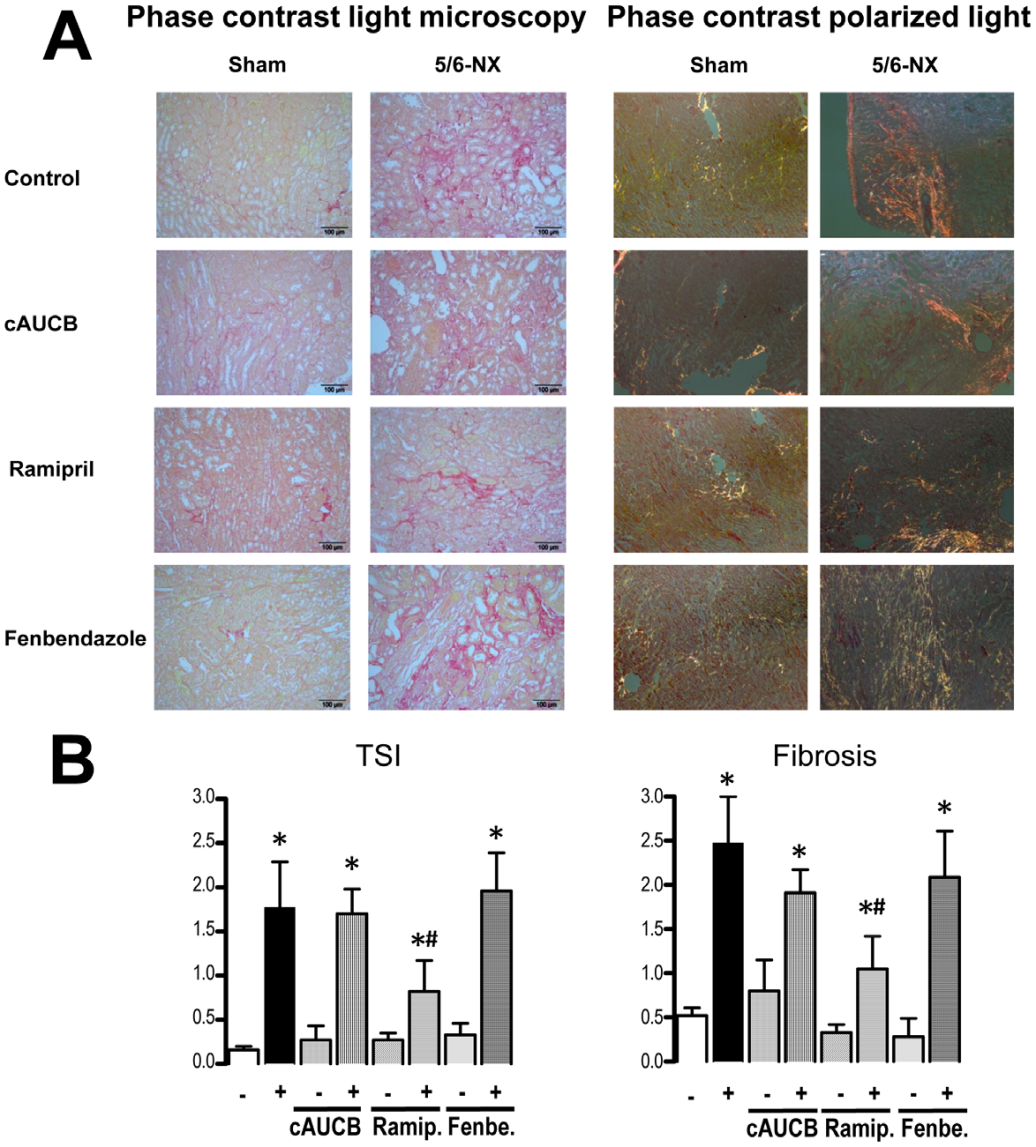
Histological tubulointerstitial changes. Representative kidney sections (magnification ×100) stained by Sirius Red in phase contrast light microscopy and polarized light microscopy scoring tubulointerstitial damage index (TSI) and interstitial fibrosis after 8 weeks of treatment. Effects of placebo, sEH-inhibition by cAUCB, ACE-inhibition by ramipril (Ramip.) and CYP-inhibition by fenbendazole (Fenbe.) are shown on sham operated (−) and 5-6-Nx (+) animals. * p<0,05 vs. sham, # p<0,05 vs. placebo (n = 5–8/group). The scale bar denotes 100 µmeter.

**Figure 4:**
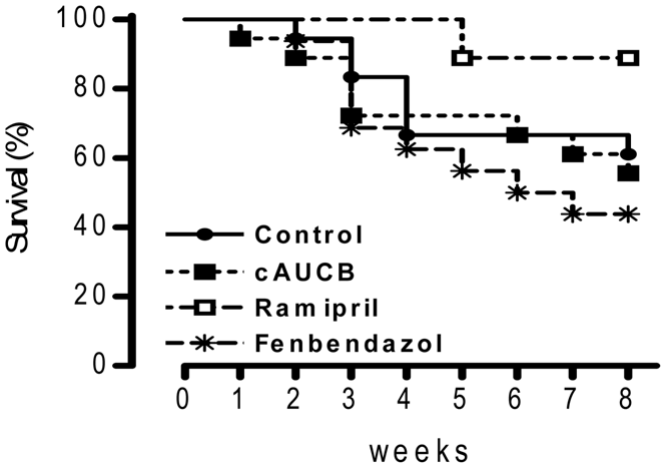
Survival data. Kaplan-Meier survival curves after 5/6-Nx in mice receiving the treatment indicated. n = 16, Survial was significantly better only in mice receiving ramipril.

To determine the extent of renal damage, semi-quantitative analyses of renal damage scores were performed on histological sections of the whole kidney. 5/6 nephrectomy lead to a significant increase in glomerulosclerosis index (**Fig. 2**) and tubulo-interstitial damage index compared to sham operated animals (**Fig. 3**), while mesangiolysis index was comparable (**Fig. 2 und 3**). cAUCB treatment had no effect on the development of glomerulosclerosis and tubulo-interstitial damage compared to the placebo treated group. In contrast, histological damage score was significantly lower in 5/6-Nx-mice receiving ramipril (p<0.05). Accordingly, late mortality in mice subjected to ACE inhibition but not to the sEH inhibitor was significantly reduced as compared to placebo-treated animals (**Fig. 4**).

### EET plasma level are elevated in the 5/6-Nx model

**Figure 5:**
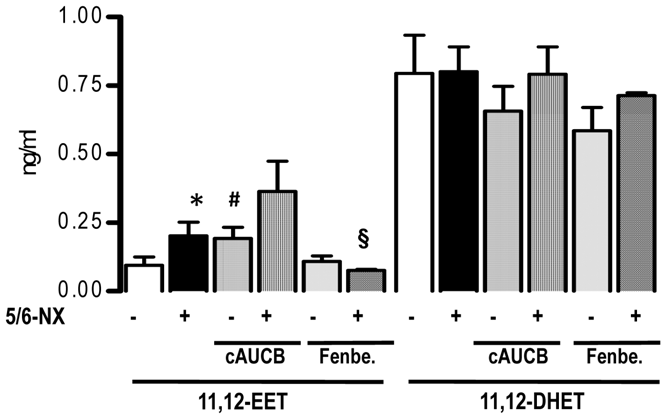
EET plasma level. 11,12-EET and 11,12-DHET plasma levels 8 weeks after initiation of treatment. Effects of placebo, sEH-inhibition by cAUCB, and CYP-inhibition by fenbendazole (Fenbe.) are shown on sham operated (−) and 5-6-Nx (+) animals. * p<0,05 vs. sham, # p<0,05 vs. placebo, § p<0.05 vs. placebo and cAUCB (n = 5–8/group).

In order to determine whether or not a possible shortage of basal EET formation after 5/6-Nx underlies the failure of sEH inhibitors to interfere with disease progression, plasma levels were measured. Animals subjected to 5/6-Nx presented with significantly increased EET plasma levels as shown for 11,12-EET (**Fig. 5**). This effect was equally strong as the increase of EETs in response to sEH inhibition by cAUCB in sham animals. Although EET levels were already elevated in 5/6-Nx mice, sEH-inhibition by cAUCB in 5/6-Nx- animals further increased the EET-levels (**Fig. 5**). ACE-inhibitor therapy had no effect on EET plasma levels (data not shown). Similar effects were seen for 5,6-EET, 8,9-EET and 14,15-EET (data not shown). No significant effects were seen on DHET plasma levels excluding product inhibition of sEH as a possible mechanism for EET accumulation in chronic renal failure. The latter observation suggest that renal function is not a limiting factor for DHET excretion and my suggest that these lipids are, potentially after conjugation in the liver, excreted via the bile.

**Figure 6:**
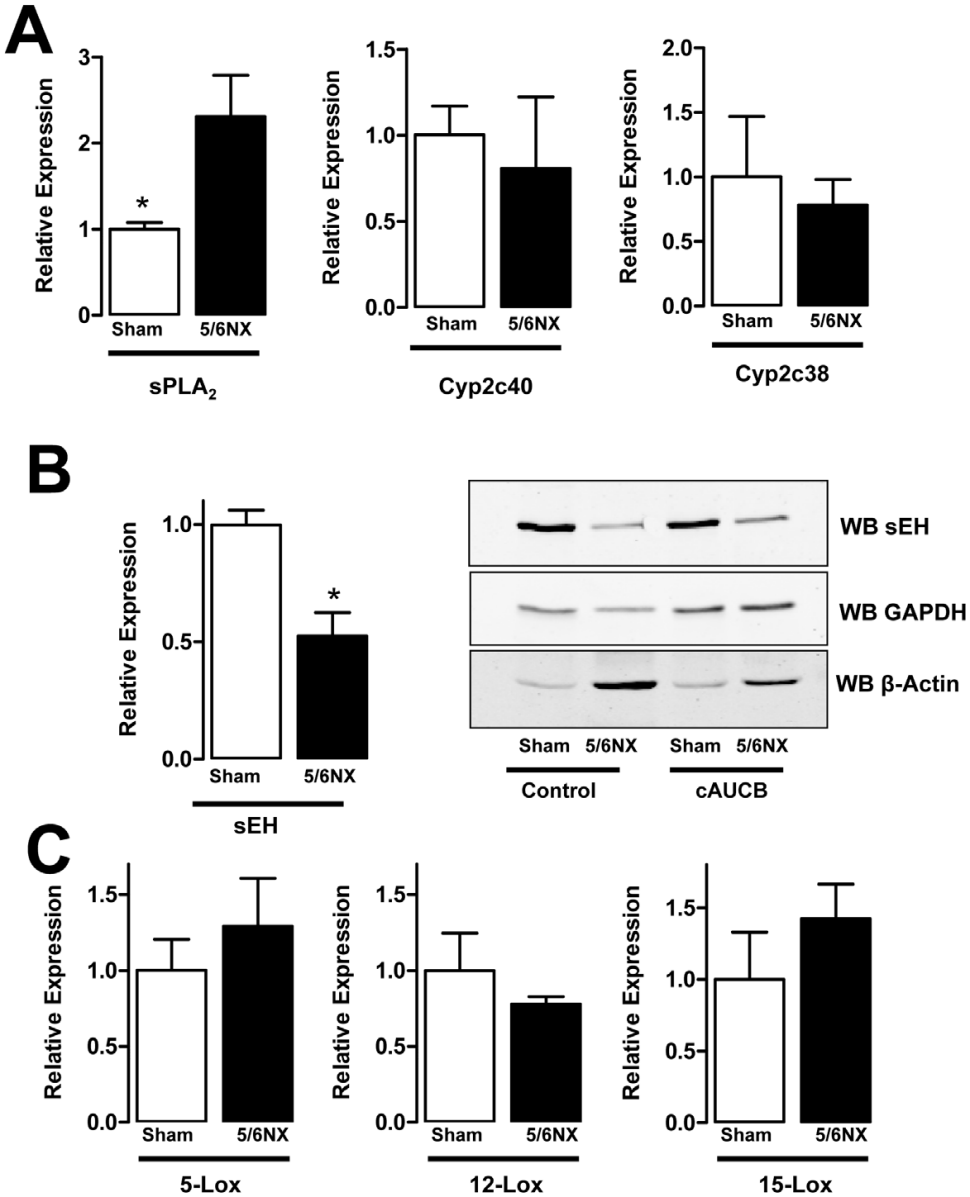
mRNA expression of arachidonic acid metabolizing enzymes. Relative mRNA expression (2∧-ΔΔCT) determined by realtime RT-PCR for the factors indicate and representative sEH Western blot (WB sEH) from the kidney of sham operated and 5/6-Nx mice at the end of the study. A: Determination of EET-producing systems, B: qRT-PCR and Western blot for sEH. GAPDH and β-actin were used as loading control for the Western blots. C: Expression of Lox enzymes on the RNA level. n = 6, *p<0.05.

In order to determine whether changes in EET production underlie the effects observed, the renal expression of sPLA2, sEH and CYP450 enzymes was determined. Whereas the expression of Cyp2c40 and 2c38 was unaffected by 5/6-Nx, sPLA_2_-mRNA expression was increased, suggesting a possible enhanced supply of CYP450-enzymes with arachidonic acid (**Fig. 6A**). Interestingly, 5/6-Nx also resulted in a marked decrease in the renal expression of the sEH on the protein, as well as on the mRNA level (**Fig. 6B**). This observation may suggest that in the diseased kidney local effects of sEH inhibitors are less pronounced than it could be assumed from the changes in plasma EET level. It also indicates that the renal sEH, although highly expressed in the kidney, only has a minor contribution to the systemic turnover of EETs.

### Inhibition of sEH and 5/6-Nx results in increased plasma level of lipoxygenase products

**Figure 7:**
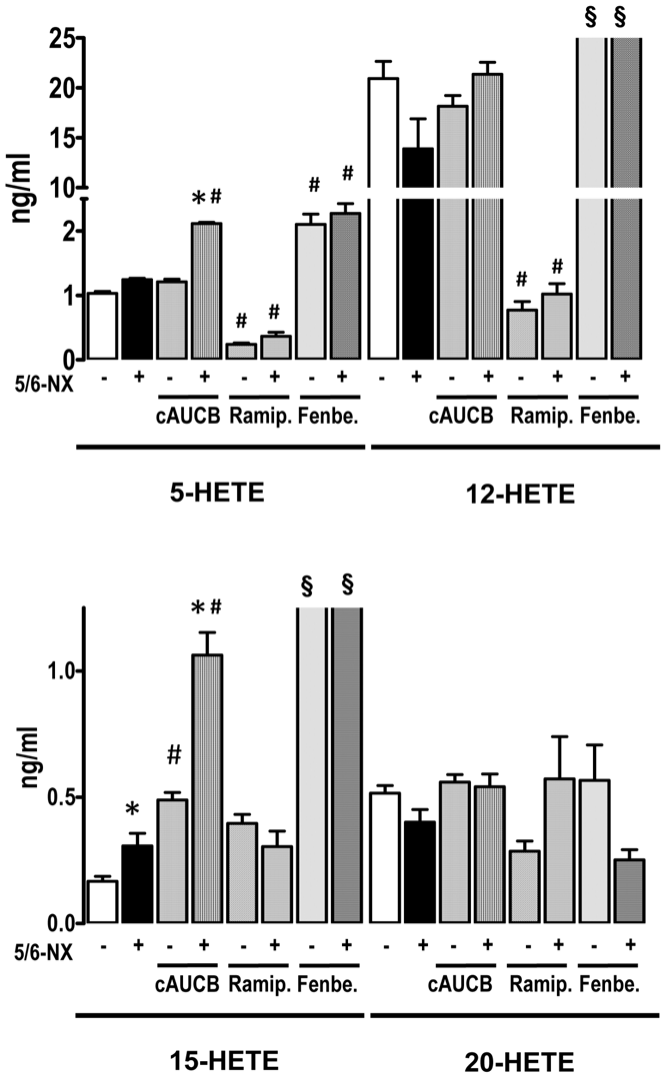
Plasma levels of hydroxyeicosatetraenoic (HETE) acids. Plasma level of 5-HETE, 12-HETE, 15-HETE and 20-HETE as determined by LC-MS/MS 8 weeks after initiation of treatment. Effects of placebo, sEH-inhibition by cAUCB, ACE-inhibition by ramipril (Ramip.) and CYP-inhibition by fenbendazole (Fenbe.) are shown on sham operated (−) and 5-6-Nx (+) animals. * p<0,05 vs. sham, # p<0,05 vs. placebo, § p<0.05 vs. placebo all groups (n = 5–8/group).

To further elucidate the lack of beneficial effects of sEH inhibition on the progression of chronic renal failure, we investigated the pro-inflammatory lipoxygenase system. 5/6-Nx had no significant effect on the renal expression of 5-Lox and 12-Lox, whereas a trend towards increased expression of 15-Lox was observed. (**Fig. 6C**) Although this did not reach statistical significance, 15-HETE plasma levels were the only lipoxygenase products which were significantly higher in the plasma of mice after 5/6-Nx receiving placebo treatment (**Fig. 7**). Interestingly, this lipoxygenase product was also increased in mice receiving sEH-inhibitor treatment and this effect was additive leading to more than 4-fold higher plasma levels in 5/6-Nx under sEH inhibitor treatment as compared to control animals not receiving treatment. Also 5-HETE was increased in 5/6-Nx mice on sEH-inhibitor treatment. In contrast, 12-HETE and the Cyp450a4-product 20-HETE were unaffected in this scenario, excluding that unspecific accumulation of lipid peroxides are responsible for the effects observed. 5-HETE and 12-HETE levels were decreased under ACE inhibitor treatment, but ACE inhibition had no effect on 15-HETE and 20 HETE levels.

### Inhibition of the CYP450 pathway in 5/6-Nx results in accumulation of lipoxygenase products

A possible explanation for the effects on the pro-inflammatory hydroxylipids is that high levels of EETs shift arachidonic acid into the LOX pathways. In order to test this hypothesis, we treated a subgroup of mice with the unspecific CYP450 inhibitor fenbendazole. Fenbendazole was effective in inhibiting CYP as demonstrated by decreased 11,12-EET (**Fig. 5**) and 20-HETE plasma levels (**Fig. 7**). Importantly, the compound had a similar effect on the progression of renal failure in 5/6-Nx mice as the sEH inhibitor: It did not affect blood pressure (**Fig. 1A**) or histological lesion scores (**Fig. 2 and 3**) but significantly increased albuminuria after 8 weeks of treatment in 5/6-Nx mice (**Fig. 1B**). Moreover, fenbendazole basically increased all lipoxygenase products measured in this study, demonstrating that under CYP-inhibition substantial amounts of AA are shifted into the lipoxygenase pathway (**Fig. 7**).

## Discussion

In this study, we determined the impact of sEH inhibition on the progression of renal failure in mice subjected to the 5/6-Nx model. In contrast to our expectations, sEH inhibition did not delay disease progression but rather resulted in an increase in albuminuria. sEH inhibition increased some lipoxygenase products and resulted in similar changes as observed with the broad-spectrum CYP450 inhibitor fenbendazole. These findings may suggest that EETs are less important in protecting the kidney than initially anticipated but that increased formation of lipoxygenase products by shifting of arachidonic acid from the CYP450 pathway into the LOX pathway might be important for the progression of the disease.

One important progression factor of chronic renal disease is hypertension. The antihypertensive effect of sEH inhibition has been demonstrated in numerous rat and mouse models, like spontaneously hypertensive rats, angiotensin II-induced hypertension and DOCA and salt-induced and salt-sensitive hypertension [7], [11], [25], [26]. The antihypertensive effect was mediated by a decrease in vascular resistance and enhanced renal Na^+^ excretion [2], [7], [10], [11], and all these resemble biological effects of EETs [25], [27], [28].

In contrast to these observations, in the present study, sEH inhibition had no effect on blood pressure in 5/6-Nx mice. The pathogenesis of hypertension in chronic renal failure is however complex: In addition to an activation of the renin angiotensin system (RAS), sodium and water retention and thus increased cardiac output contribute to the situation [13], [14], [29]. The contribution of RAS to the hypertension thereby is not easy to estimate. Although the ACE inhibitor ramipril prevented the development of hypertension in the present study, it also interfered with disease progression, making it highly probable that also sodium and water retention were reduced by ramipril [30]. Moreover, the compound had a natriuretic effect on its own and promotes sodium excretion by multiple ways.

It should also be mentioned that the antihypertensive effect of sEH inhibitors is model dependent and not observed in stroke prone SHR [31] and Goto-Kakizaki rats [32] and of a variable degree in the different strains of SHR [31], [33], [34]. Also conflicting results have been published in sEH ^−/−^ mice, in which the antihypertensive effect was lost after backcrossing on the C57/BL6 background [12], [30], [35]. Although blood pressure lowering effects of sEH inhibition depend on their vasodilative and natriuretic effects, it is also possible that the renal dysfunction after 5/6-Nx [29] attenuates the efficacy of sEH inhibition on blood pressure.

As one would expect, sEH inhibition-mediated lowering in blood pressure exerts reno-protective effects in models of hypertensive kidney injury [10]–[12]. In hypertensive models in which sEH inhibitors failed to lower the blood pressure this reno-protective effect was observed, too [32], [35]. Also the acute renal injury by the tubulotoxic chemotheraptic agent cisplatin was attenuated by sEH inhibitors [36]. The latter three studies had in common that sEH inhibition lowered several markers of inflammation, another progression factor for renal disease.

The data of the present study demonstrated that sEH inhibition in 5/6-Nx rather increased than decreased disease progression. In keeping with a possible role of inflammation, we could demonstrate that lipoxygenase products, particularly of the 15-LOX pathway, but also of the 5-LOX pathway accumulated in 5/6-Nx mice treated with sEH inhibitors. The biological effects of the two LOX enzymes differ to some extent. 5-LOX is considered pro-inflammatory in general [37] and 5-LOX inhibitors exerted reno-protective effects [38]. 15-LOX in contrast, not only generates pro-inflammatory lipids but 15-HETE is further metabolized to lipoxins, which have strong anti-inflammatory properties [39]. On the other hand, inhibition of 15-LOX had beneficial effects on diabetic models [40] or renal injury and mesangial cells of 12/15-LOX knockout mice had a reduced level of activation after stimulation, which was associated with reduced matrix production [41]. Indeed, it is important to remember that chronic progressive renal failure is a complex disease with a strong fibrotic contribution. Interestingly, a strong positive interplay exists between 15-LOX-products and the pro-fibrotic transforming growth factor β (TGF β)-pathway [42]. One limitation of all these studies, however, is that they are predominantly performed in glomerular cells or culture and that therefore the role of LOX for tubulo-interstitial fibrosis has to remain unclear. Thus, also a limitation of the present study is that we did not determine the effect of sEH inhibition in animals lacking LOX activity and therefore we cannot provide definite proof for an involvement of LOX in the progression of renal failure after sEH inhibitor treatment.

A potential explanation for the observations of the present study is that sEH inhibition shifted arachidonic acid from the CYP450 pathway into the LOX pathway. That such shifting occurs is well known for COX inhibitors and the basis of NSAIDS-induced asthma [43]. Direct inhibition of CYP450 with the compound fenbendazole [44] also increased the products of the LOX pathway, demonstrating that shifting of arachidonic acid also from the CYP450 pathway can occur. Given the low plasma concentrations of EETs, it is however difficult to imagine that the somewhat modest increase in response to sEH inhibition or renal failure is sufficient to induce a significant product inhibition of the CYP enzymes, although other observations supporting the complex crosstalk have been published for inflammatory models [45]. An alternative explanation could therefore be that the increase in sPLA_2_ mRNA observed in this study results to in overall increase in all arachidonic acid pathways. Unfortunately, the amount of plasma samples obtained in the course of this study did not allow us to analyze the lipidom in more detail to further clarify this point. We therefore also did not measure the different enantiomers of the HETEs to definitely prove that the increase of this hydroxylipids is a consequence of the action of LOX and not of lipid peroxidation.

In this study we observed that the sEH expression decreases during progressive renal failure. As this effect occurred on the message as well as on the protein level, it is unlikely that changes in the matrix composition which occur during the fibrotic remodeling of the kidney could account for this observation. Interestingly, also in the lung we previously observed that fibrosis occurring during pulmonary hypertension reduced the sEH expression [9] suggesting that this effect is more important than the previously reported induction of sEH by angiotensin II [46]. The fact that the relative increase in plasma EET level were similar between normal mice and those in renal failure suggest that the renal sEH contributes little to the EET plasma level. The increase in EET plasma level in response to 5/6-Nx is either a consequence of a reduced renal excretion or of a greater formation of EET under this condition.

Independently of the mechanism of increased EET plasma level, the present study demonstrates that lowering sEH activity is not necessarily beneficial. Importantly, this notion is also supported by data on human polymorphisms: In kidney transplanted patients a gene polymorphism leading to reduced sEH activity was linked to allograft dysfunction and decreased graft survival [47].

In conclusion, in the present study we demonstrate that sEH inhibition rather promotes than delays the progression of chronic renal failure in mice. As a possible mechanism we suggest the accumulation of inflammatory lipoxygenase products. Thus, chronic renal failure as a possible drug target for sEH inhibitors should be reconsidered.
